# A75 AUTOMATED FOLLOW-UP USING A PATIENT-GUIDED COMPLICATION TRACKING SYSTEM (PACTS): AN UPDATE ON PROGRESS

**DOI:** 10.1093/jcag/gwab049.074

**Published:** 2022-02-21

**Authors:** L Calman T’ien, C Galorport, J J Telford, R A Enns

**Affiliations:** The University of British Columbia Faculty of Medicine, Vancouver, BC, Canada

## Abstract

**Background:**

In recent years, there has been an increase in automated interventions in medicine. The COVID-19 outbreak has further fueled this rise. In response to the pandemic, Healthcare systems have developed a multitude of technological strategies for case identification and contact tracing. It is in this evolving digital landscape, that a PAtient-guided Complication Tracking System (PACTS) was launched.

PACTS allows clinics to track complications using the Short Message Service (SMS). This program also offers opportunities to augment medical services and support patients having complications.

Before PACTS can be widely implemented in clinics, research needs to be conducted to investigate its potential as a complication tracking software.

**Aims:**

To assess the outcomes of an automated follow-up program implemented at St. Paul’s Hospital in Vancouver, BC.

**Methods:**

A prospective study was designed to contact outpatients one-week post-procedure using PACTS.

This program was delivered in two phases. Stage 1 ran from November 2019-March 2020. During this pilot stage, patients having a colonoscopy or gastroscopy were asked to participate in the study. Stage 2 ran from August 2020-August 2021. For this phase, patients having a colonoscopy, gastroscopy or flexible sigmoidoscopy were automatically enrolled in the study. An independent t-test was completed to assess response rate differences between stages.

SMS responses were recorded and patients having unplanned events were contacted by phone to categorize complications. Adverse events (AE) were defined as side-effects requiring telehealth follow-up or emergency room visitation. Severe adverse events (SAE) were classified as complications requiring admission to hospital (>24 hrs).

**Results:**

SMS prompts were sent to 6975 patients and the overall mean response rate was 89%. The mean response rates from Stages 1 and 2 were 92% and 88% respectively. The independent t-test revealed a statistically significant difference in response rates between phases, two-sample t(174) = 4.56, p = 9.58 x 10^–6^.

498 (8%) of SMS respondents reported having unplanned events. Of these patients, 372 (75%) were reached by phone and 257 (69%) were confirmed to have had a side effect. 65 of these complications were AEs and of these, 3 cases were SAEs. The most common AEs were abdominal pain (37%), bleeding (35%), nausea and vomiting (14%).

**Conclusions:**

The high response rates achieved during this study provide further evidence for the use of automated follow-up systems in medicine. This study also demonstrates the potential of PACTS as a complication tracking software.

Future research should devise strategies to optimize the collection of complication data using an SMS-based service.

**Funding Agencies:**

None, NRC

**Table:** Overview of SAEs Captured by the PACTS Program.

SAE ID	Initial Procedure	Reason for Admission	Post-procedural Intervention
1	Colonoscopy	Bleeding	Blood transfusion & colonoscopy with clip placement
2	Colonoscopy	Bleeding	Blood transfusion & gastroscopy
3	Gastroscopy	Bleeding & perforation	Blood transfusion & antibiotics

